# Clinical characteristics of protracted bacterial bronchitis in Chinese infants

**DOI:** 10.1038/srep13731

**Published:** 2015-09-04

**Authors:** Yuqing Wang, Chuangli Hao, FanFan Chi, Xingmei Yu, Huiquan Sun, Li Huang, Meijuan Wang, Wei Ji, Yongdong Yan, Hong Zhu, Xuejun Shao

**Affiliations:** 1 Department of Respiratory Medicine, The Affiliated Children’s Hospital, Soochow University, Jingde Road No. 303, Suzhou 215003, China; 2 Department of Laboratory Medicine, The Affiliated Children’s Hospital, Soochow University, Jingde Road No. 303, Suzhou 215003, China

## Abstract

Protracted bacterial bronchitis (PBB) is the common cause of chronic cough in children worldwide, but its etiology has not been fully recognized in China. We retrospectively investigated a total of 66 hospitalized infants under the age of three years with chronic wet cough enrolled in the Affiliated Children’s Hospital of Soochow University from October 2010 to March 2014. All patients underwent bronchoscopy and broncho-alveolar lavage (BAL) samples were processed for microbiological and cytological analysis. Of 66 patients with wet cough, 50 (75.8%) were diagnosed with PBB. In the PBB group, wet cough was accompanied by wheezing (90%). Airway malacia were identified in 22 cases (44%). The clinical manifestations of PBB with airway malacia did not differ from those without malacia. Haemophilus influenzae (47.4%) and Streptococcus pneumoniae (36.8%) were the most commonly identified pathogens. Furthermore, CD3^+^ and CD3^+^CD4^+^ cells were significantly lower in the PBB group (p < 0.01), while CD19^+^, CD16^+^CD56^+^ and CD23^+^ cells were elevated (p < 0.01) in the PBB group. Our study revealed PBB is an important cause of chronic wet cough in Chinese infants, and that changes of lymphocyte subsets are observed in children with PBB. Airway malacia frequently co-existed with PBB, but did not exacerbate the disease.

In China, at least 10% of patients visit their general practitioner for treatment of chronic cough^1^. Chronic cough which lasts longer than four weeks is one of the most common symptoms in children referred to a hospital. The causes of chronic cough in children differ from the causes in adults and vary according to different age groups and areas^2^. A multi-center study on the cause of chronic cough in children in China found that the majority of causes were cough variant asthma (CVA) (41.9%), upper airway cough syndrome (UACS) (24.7%), and post-infection cough (PIC) (21.7%)^3^. Marchant *et al.* reported in an Australian study that the most common cause (40%) of chronic cough in children was protracted bacterial bronchitis (PBB)^4^. Asiloy *et al.* studied 108 children aged between six months to 14 years with chronic cough in Turkey and PBB accounted for 23.4% of the cases^5^.

PBB as a major cause of chronic cough in children has officially been recognized by the British Thoracic Society, the Thoracic Society of Australia and New Zealand^6^,^7^. PBB is associated with persistent or protracted bacterial infection of the respiratory airways. Haemophilus influenzae, especially non-typable H. influenza strains, Streptococcus pneumoniae and Moraxella catarrhalis are the three most commonly identified bacteria^8^. The occurrence of PBB is related to bacterial biofilm formation in the airway, impaired mucociliary clearance, systemic immune function defects, and airway anomalies^9^,^10^.

The original diagnostic criteria for PBB includes (a) wet cough >four weeks duration, (b) identifiable lower airway bacterial infection on broncho-alveolar lavage (BAL) culture, and (c) response to antibiotics (amoxicillin/clavulanate) with resolution of cough within two weeks, (d) the absence of an alternative specific etiology^6^,^7^. If left untreated, PBB may develop into chronic suppurative lung disease (CSLD) in some children and possibly bronchiectasis^8^. Despite the fact that PBB is known to constitute an important cause of chronic cough in young children, studies on PBB characteristics are rare, and little is known about the etiology of PBB in children in China. PBB is often misdiagnosed as bronchial asthma or bronchial pneumonia, because pediatricians lack awareness of the disease. The aim of this study was to describe the clinical characteristics of PBB among patients under the age of three years with chronic wet cough in China.

## Results

### Demographic and clinical characteristics of patients with PBB

A total of 66 patients were enrolled in this study, and 50 patients were diagnosed with PBB; 43 (86%) boys and seven (14%) girls (Table 1). The proportion of males was higher than that of females (p < 0.05). The youngest patient was two months old, and the oldest patient was two years and 11 months of age. The median age was 10 (5.75–14) months. Twelve patients (24%) were younger than six months of age, 19 (38%) were six months to one year of age, and 19 (38%) were one to three years old. No statistical differences were observed between the age or gender groups (p > 0.05). Children with chronic cough due to other causes was confirmed according to standard clinical practice definitions [7], and those who did not fulfill the criteria for PBB were stratified into the ‘no PBB group’ (n = 16). The diagnoses in this group were bronchial asthma (n = 5), upper airway cough syndrome (n = 5), post-infection cough (PIC) (n = 3), Bordetella pertussis-like symptoms (n = 2), and gastroesophageal reflux (n = 1).

In the PBB group, there was variation in severity of wet cough, including five cases (10%) of simple wet cough, two cases (4%) of wet cough accompanied by stridor, and 43 cases (86%) of wet cough accompanied by wheezing (Table 1). The median duration of cough was 10.0 ± 1.6 weeks, the shortest duration was four weeks, and the longest duration was recorded as 96 weeks. For eight cases (16%) the phase of cough was in the morning, eight cases (16%) of coughing were in the night, two cases (4%) showed post-activity cough, and 32 cases (64%) were coughing both day and night. Wheezing and/or moist rales were recorded in 48 cases (96%). Patients in the PBB and the ‘no PBB’ groups presented most clinical symptoms equally. There was no significant statistical difference in the median duration of cough between the two groups. However, the presence of day and night cough (64% vs. 43.8%, p < 0.05) and wheezing (90% vs. 68%, p < 0.001) were statistically more likely in children with PBB (Table 1). All of the patients received X-ray examination of the chest. Abnormalities of chest radiography were found in 26 cases (52%), including 24 (48%) cases with increased and blurred bilateral lung markings, and two cases (4%) with patchy shadows.

### Bronchoscopic findings, demographic and clinical features of PBB patients with or without airway deformity

**Figure 1:**
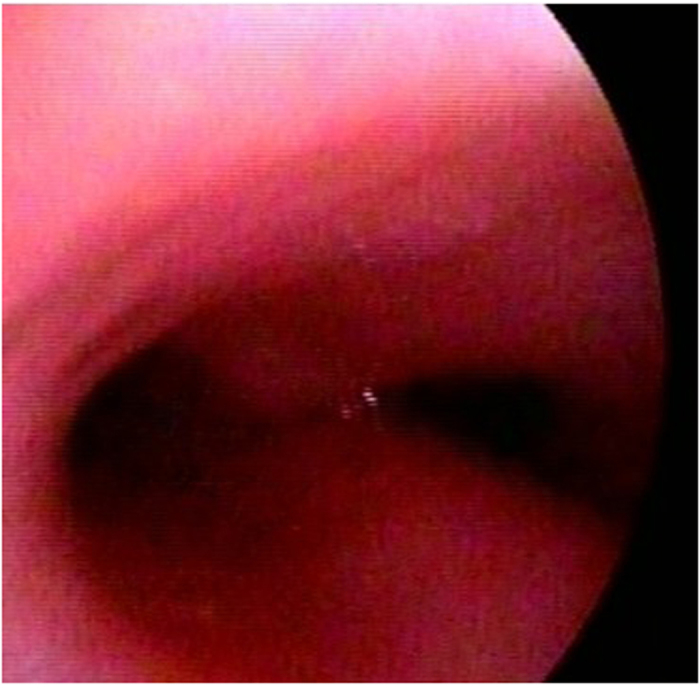
Bronchoscopic findings of a patient with PBB. Redness and edema of the bronchial mucous membranes accompanied by bronchomalacia are seen under bronchoscopy.

Bronchoscopy of the 50 patients with PBB revealed redness and edema of the bronchial mucous membranes as the main characteristic. Thirty-three cases (66%) had thin secretions, eight cases (16%) had sticky secretions, six cases (12%) had funicular secretions, and three cases (6%) had phlegm blots. Airway deformities were identified in 22 cases (44%), including: seven laryngomalacia (31.8%), three tracheomalacia (13.6%), nine bronchomalacia (40.9%), two laryngo- and bronchomalacia (9.1%), and one tracheobronchomalacia (4.5%) (Fig. 1).

When patients with PBB were examined, immune function, cellularity and bacterial culture results of BALF showed no significant differences, except that patients with airway malacia were younger than those without malacia (8 month, range 5–12 months) vs. 12 month (range 7.25–16.75 months), p < 0.05). Stridor was more common in patients with airway malacia (Table 2).

### Cytology, bacteriology and immune status in PBB patients

Cytology examination of BALF (Table 3) showed that the median percentage of neutrophil cells was markedly elevated (29.2% ± 30.2, p < 0.01) in children with PBB compared to the ‘no PBB’ group (2.9% ± 3.5). The number of macrophages was significantly decreased in children with PBB (61.0% ± 32.7, p < 0.01) compared with the ‘no PBB’ group (88.7% ± 3.6). There was no significantly statistical difference in the median percentage of lymphocytes and eosinophil cells observed between PBB and the ‘no PBB’ group (Table 3).

Positive bacterial cultures were identified in 19 of 50 specimens (38%), and none of the control patients had a positive bacterial culture (Table 4). Haemophilus influenzae was found in nine cases (47.4%), Streptococcus pneumoniae in seven cases (36.8%), E. coli in two cases (6.1%), and Enterobacter aerogenes in one case (5.3%).

### Immune function

No statistical differences in the levels of IgA, IgG and IgM were observed between PBB and the ‘no PBB’ group. However, measurement of the lymphocyte subsets revealed that the percentage of CD3^+^ and CD3^+^CD4^+^ T cells were significantly lower in the PBB group compared to the ‘no PBB’ group (p < 0.01). In contrast, the percentages of CD3-CD19^+^, CD3-CD16^+^, CD56^+^ and CD19^+^CD23^+^ lymphocytes were significantly elevated (p < 0.05*, p < 0.05* and p < 0.01**, respectively; Table 3). No statistically significant associations between level of lymphocyte subsets and cough severity or duration were observed in the PBB group (p > 0.05, Tables 4 and 5).

### Outpatient treatment of patients with PBB before hospitalization

Most PBB patients received outpatient treatment for chronic cough symptoms before hospitalization, including six patients (12%) who received bronchial asthma treatment (inhaled corticosteroids), and 37 patients (74%) receiving antibiotics. Twenty three patients were given cephalosporin (median duration 6.2 days), five cases were prescribed azithromycin (median duration 3.8 days), and only two cases were taking amoxicillin and clavulanate (median duration 5 days).

### Treatment of patients and follow-up

In the hospital, all patients received oral antibiotic treatment including amoxicillin and clavulanate (30 mg/kg/time, three times a day). The average duration of antibiotic treatment was 17.4 ± 9.0 days with the longest duration being 22 days. All patients had outpatient follow-up every week for a total of four weeks. Verbal category descriptive (VCD) cough scores were 6.07 ± 1.56 before medication commencement. Thirty-eight cases (76%) showed resolution of cough within the two weeks of medication. VCD cough scores were1.60 ± 0.95.

## Discussion

Chronic cough is very common in Chinese children^11^,^12^,^13^. Wurzel *et al.*^14^ reported that PBB, characterized by protracted wet cough >4 weeks and bacterial culture-positive BALF, which resolved with antibiotic treatment, is the most common cause of chronic cough in children. Donnelly *et al.*^15^ retrospectively analyzed 81 cases of children with PBB, and found that two weeks of amoxicillin/clavulanic acid treatment is an effective anti-microbial treatment. In the present study, we found PBB in three quarters of children less than three years old with chronic wet cough, indicating that PBB is also an important cause in children with chronic wet cough in Suzhou, China. The proportion of PBB in children with chronic cough was higher than that found in previous reports^4^,^5^. This may be related to the relatively small sample size due to the fact that the patient number in our study was limited to children with chronic wet cough whose parents agreed to bronchoscopy.

The original diagnostic criteria for PBB included the presence of lower airway bacterial infection. We diagnosed PBB according to the following criteria: 1) Chronic isolated, wet cough which was present for >4 weeks without any clinical findings suggestive of an alternative diagnoses and resolution with appropriate antibiotic treatment for two weeks^16^,^17^,^18^,^19^. 2) The Guideline for diagnosis and treatment of PBB in Chinese children: (a) isolated wet cough for >4 weeks; (b) positive broncho-alveolar lavage (BAL) culture or neutrophils >3.5% in BAL; and (c) resolution of cough with antibiotic treatment within two weeks; (d) the absence of alternative etiology^20^. 3). Riedler and colleagues reported broncho-alveolar lavage cellularity in healthy children. The normal range of Neu % is 0.6 ± 3.5, with neutrophils >3.5% in BAL indicating neutrophilic inflammation^21^. In this study, patients with PBB had wet cough for more than four weeks with no signs of acute infections yet no other pathogens were found. The cough was resolved with antibiotic treatment within two weeks. Therefore, we diagnosed PBB. The reason that bacteria were detected in only 38% of children might be that the majority of patients were treated with antibiotics in the clinic, which will influence the result of the bacterial culture. However, the use of antibiotics was less than one week initially and most likely insufficient to treat PBB.

Previous studies have suggested that PBB occurred mainly in younger children under three years, was predominant in males, and that PBB co-exists with airway malacia^9^,^22^ . The data obtained in this study are consistent with these reports. The common symptoms of PBB include wet cough, wheezing, and stridor. Pulmonary signs of moist rales and/or wheezing was present in many cases. Regarding the phase of cough, there was no difference between day and night in those patients with PBB, although there was a difference between the diagnosis of CVA and UACS. Cough caused by CVA often occurs during the night and/or in the early morning, and after exercise. Cough related to UACS is usually caused by changes with the body position or occurs in the morning^6^,^7^. A high proportion of wheezing episodes was reported in children with PBB^18^. Similarly, in our study 86% of children with PBB presented with wheezing, which may cause pediatricians to misdiagnose PPB as bronchial asthma. Making an accurate diagnosis of asthma in children less than 3 years of age is difficult for some inexperienced pediatricians. We have diagnosed asthma according to the guideline for the diagnosis and optimal management of asthma in children^23^. Bronchial asthma is diagnosed by episodic wheeze and cough demonstrated by bronchodilator responsiveness, and/or response to inhaled steroids with resolution of cough within the first two weeks of treatment; no response to antibiotic treatment; the patient usually has atopy and a family history of asthma; and other disease-causing wheezing is excluded.

Five (12%) of our cases were misdiagnosed as asthma but did not respond to inhaled corticosteroid treatment. Several studies^24^,^25^,^26^ found that the co-existence of PBB with airway malacia and neutrophilic inflammation in the BAL had a strong correlation with wheezing. Marchant *et al.*^24^ reported that children with PBB had marked airway neutrophilia and increased median inflammatory cytokine levels when compared to those with cough that resolved naturally or those with no cough at all. Le Bourgeois *et al.*^25^ reported that neutrophil-mediated inflammation in the airways appears to better characterize recurrent wheezing in children < three years old. De Schutter *et al.*^27^ found high rates of bacterial infection and marked neutrophilic inflammation of young children with persistent wheezing. These data provide a possible explanation for the non-response to inhaled corticosteroid therapy. Thus, when treating children with chronic wet cough accompanied by wheezing who do not respond to inhaled corticosteroid treatment, clinicians should consider PBB as a possible precipitant and perform a bronchoscopy.

In our study, congenital airway malacia, including laryngomalacia, tracheomalacia, bronchomalacia, laryngo- and bronchomalacia, tracheo- and bronchomalacia, was found in 22 cases (44%) with PBB. The detection rate of malacia was higher than that observed in previous reports^28^. Kompare *et al.* reported that airway malacia was presented in 74% of children with PBB^26^. The authors hypothesize that airway malacia is a predisposing anomaly for PBB. Airway collapse decreases effectiveness of cough and can interfere with normal cephalad mucous flow^29^. The presence of tracheomalacia may impair mucus clearance and cause retention of tracheobronchial secretions, which facilitates persistence of lower airway infection. Comparing PBB with airway malacia to PBB with no malacia, we found that children with airway malacia were younger than those without malacia. The clinical manifestation, immune function, cellularity and bacterial culture of BALF from patients with airway malacia did not differ from those without malacia. Our results suggest that although airway malacia was frequently observed in children with PBB, co-existing airway malacia did not aggravate the disease.

In the present study, we evaluated the immune function of patients with PBB. Although basic immunoglobulin levels were normal, abnormal differences in lymphocyte subsets were observed between the PBB and the ‘no PBB’ group. The percentage of CD3^+^ and CD3^+^CD4+ cells were significantly lower in the PBB group compared to the control group. In contrast, the percentage of CD19^+^, CD16^+^CD56^+^ (natural killer, NK) cells, and CD23^+^ cells was significantly elevated. The changes of lymphocyte subsets in children with PBB suggest the existence of an immune disorder, which may be related to the occurrence of PBB. When T cell subsets and cough severity and duration were analyzed in PBB patients to assess if there was a significant correlation, we could not demonstrate such an association, indicating that the observed changes in T-cell levels did not exacerbate the disease. Decreased numbers of CD3^+^ and CD3^+^CD4^+^ helper T lymphocytes affect the body’s ability to remove pathogens and are likely associated with persistent bacterial infections in the lower airway and the prolonging of disease. CD19^+^ cells, representing activated B cells, can mediate an immune response by secreting a variety of immunoglobulins. It could be considered that the bacterial infectious aspect of PBB and the concomitant immune response causes CD4^+^ auxiliary T-lymphocyte activation, which in turn leads to the activation of antibody-producing B cells. Wurzel DF *et al.*^14^ reported that elevated CD16^+^ and CD56^+^ (NK) cells in children with PBB are often related to elevated virus detection rates in BAL. NK cells play a role in innate immune defense against viral infections. However, we did not investigate respiratory viral infections in BALF. The reason for high NK cell levels in children with PBB is still uncertain. The mechanism linking PBB to a potential immune disorder needs to be studied further.

In the present study, an increased percentage of neutrophil cells, and reduced percentage of macrophages was observed in the BAL specimens of children with PBB. These results are consistent with other reports^4^,^22^,^30^. The most common pathogens cultured from BAL were Haemophilus influenzae (47.4%) and Streptococcus pneumoniae (36.8%), which is consistent with previous studies^4^,^14^. Zgherea *et al.* studied 109 children with chronic wet cough and obtained *H. influenzae* and *S. pneumoniae* from BAL in 49% and 20% of isolates, respectively^28^. Marchant *et al.* reported that *H. influenzae* (47%) and *S. pneumoniae* (35%) were the most frequently detected bacterial pathogens in PBB^31^. Our results, along with these studies suggest that neutrophilic inflammation is present in PBB and may be linked to the persistence of the underlying airway infections. In some places in China, there is no vaccination program for children available, which would protect against these two bacterial pathogens. Especially given the increase in antibiotic resistance, a vaccination program against *H. influenzae* and *S. pneumoniae* should be recommended for children.

The current international clinical guidelines recommend antibiotic treatment as appropriate management for PBB^6^,^8^. Marchant *et al.* reported that a two-week amoxicillin/clavulanate regimen will achieve cough resolution in a significant number of children with PBB^32^ . Donnelly *et al.* reported that 51% of the patients were completely symptom free after two courses of antibiotics, and 13% of patients required more than six courses of antibiotics^15^. In our study, 74% of patients received antibiotic therapy before hospitalization. Inadequate treatment regimens and improper selection of antibiotics could explain why the treatment failed. Upon hospitalization, all patients received antibiotic treatment, including amoxicillin/clavulanic acid for an average duration of 17.4 ± 9.0 days. Thirty-eight cases (76%) showed resolution of cough within the two weeks of medication, indicating a good prognosis for PBB. Some studies speculated that untreated PBB and/or recurrent episodes of PBB will progress to CSLD and bronchiectasis. However, the proportion of PBB developing CSLD and bronchiectasis is unknown. More long-term cohort studies are needed in the future to fully confirm this hypothesis.

The present study has the limitation that we did not investigate common respiratory viruses in BALF. Therefore, the role of viruses in coughing and wheezing has not been addressed. In addition, the time of follow-up was relatively short and it is therefore difficult to come to a definitive conclusion regarding the outcome of PBB. Further studies including virus detection and longer follow-up periods are required.

In summary, our study provides evidence that PBB is an important cause of chronic wet cough in children younger than three years of age in Suzhou, China. Clinical characteristics of PBB included wet cough which was accompanied by wheezing in most patients. Airway malacia were frequently observed in children with PBB, yet co-existing airway malacia did not exacerbate the disease. The most common pathogens cultured from the BAL from patients were Haemophilus influenzae and Streptococcus pneumoniae. Moreover, changes in lymphocyte subsets were observed in children with PBB.

## Methods

### Approvals

All experiments were performed following the relevant guidelines and regulations of Soochow University. The methods were carried out in accordance with the approved guidelines. The study was approved by the Medical Ethics Committee of Soochow University (No. Sdfey201075). The parents of all study participants gave both verbal and written informed consent before study enrollment.

### Patients

This study was retrospectively conducted in 66 patients (age < three years) hospitalized in the Department of Respiratory Disease of the Affiliated Children’s Hospital, Soochow University from October 2010 to March 2014. Fifty patients were diagnosed with PBB and 15 children did not have PBB (no PBB group). All patients had a cough for more than 4 weeks duration without acute lower respiratory tract infection (e.g. fever, shortness of breath or dyspnea, and cyanosis) and had not responded to conventional treatment. Patients with congenital heart disease, immune deficiency, bronchus or pulmonary dysplasia, neuromuscular disease, or foreign body aspiration were excluded from the study.

Demographic characteristics (age, gender, number of siblings), clinical manifestations (cough characteristics, such as phase and duration of wet or dry cough), physical examination findings, imaging results (chest X-ray, computed tomography), and treatment history were collected upon enrollment. Follow-up information on response to antibiotics and prognosis was collected by outpatient consulting and telephone interview every week, for a total of four weeks. The diagnostic criteria for PBB included: (a) isolated wet cough for >4 weeks; (b) positive broncho-alveolar lavage (BAL) culture or neutrophils >3.5% in BAL; and (c) resolution of cough with antibiotic treatment within two weeks; (d) the absence of alternative etiology^16^,^17^,^18^,^19^,^20^,^21^.

### Data collection of cough score

Standardized data collection sheets were used for all assessments. The parents completed a daily cough diary for 28 days post enrolment to document presence and severity of cough. A validated cough diary using the verbal category descriptive (VCD) score was used^32^. This involves scoring the cough as listed below for each day: 0 = no cough, 1 = cough for one or two short periods only, 2 = cough for more then two short periods, 3 = frequent coughing but does not interfere with school and other activities, 4 = frequent coughing which interferes with school and other activities, 5 = cannot perform most activities due to severe coughing. Cough resolution, defined as an improvement in baseline cough score (>75% reduction in cough score)^33^.

### Bronchoscopy

All patients underwent bronchoscopy with broncho-alveolar lavage fluid (BALF) collection. All procedures were videotaped, and the bronchoscopic findings were carefully documented. Laryngomalacia was defined as an inward collapse of supraglottic structures in the glottis on inspiration, causing airway obstruction. Tracheomalacia was defined as tracheal deformity at the end of expiration, maintained during spontaneous respiration but which could be altered by the passage of the bronchoscope or positive airway pressure. Bronchomalacia was defined as an appearance of deformity in the right or left main-stem bronchi and/or their respective divisions at the lobar or segmental level^34^. The degree of tracheomalacia and/or bronchomalacia can be divided into mild (less than 1/3 of tissue, which was invaginated), moderate (1/3 ~ 1/2 invagination), severe (more than 4/5 invagination) types on the basis of the trachea and/or bronchial tube cavity invagination. For BALF collection, the bronchoscope was inserted into the bronchus and three to four 10 mL aliquots of normal saline solution were instilled and immediately aspirated through the bronchoscope. In infants less than one year, 5 ml aliquots were used as per routine procedure at the Bronchoscopy Center of the Affiliated Children’s Hospital, Soochow University. The BALF samples were transported to the Department of Laboratory Medicine within 30 minutes.

### Microbiological and cytological analysis

BALF samples were processed for microbiological and cytological analysis. Quantitative cultures for common aerobic and anaerobic bacteria were obtained. One hundred consecutive cells were counted at 1000 × microscopic magnification, and the percentage of macrophages, neutrophils, lymphocytes and eosinophils was analyzed. The normal reference values were: macrophages 80–95%, neutrophils *<3.5%, lymphocytes* <15%, eosinophils <1%^35^. Bacterial counts ≥10^4^ colony-forming units (CFU)/ml or neutrophils >3.5% in BALF were considered positive^1^,^6^.

### Blood analysis

Blood samples were collected for basic immune function tests, including immunoglobulin (IgG, IgA, IgM) and lymphocyte subset analyses. Immunoglobulins were measured by immune turbidimetry (ORION Diagnostica, Espoo, Finland) using a HITACHI 7600 automatic biochemical analyzer. The lymphocyte subset analysis was performed from venous blood samples. Red blood cells were lysed, and the remaining lymphocytes were washed twice with PBS. If the cell count was >10 × 10^9^ cells/l, the sample was adjusted to 5 × 10^9^ cells/l in PBS. The following antibodies were added and incubated for 15 min at room temperature: phycoerythrin-Cy5 (PE-Cy5)-tag anti-CD3^+^ and anti CD19^+^ antibody, fluorescein isothiocyanate (FITC)-tag anti-CD4^+^ antibody, p-phycoerythrin (PE)-tag anti-CD8^+^ antibody, CD16^+^CD56^+^antidody, CD23^+^ antibody, and fluorescence-labeled monoclonal antibodies (CD3^+^, CD4^+^, CD16^+^, CD19^+^, CD23^+^ and CD56^+^ . The blood cell samples were analyzed by flow cytometry using an EPICS Altra flow cytometer (Beckman Coulter, Indianapolis, USA). The flow cytometry data were expressed as a percentage of the total lymphocyte population.

### Statistical analysis

All data were analyzed using SPSS/PASW 20.0 statistical software (IBM, Armonk, NY, USA). Student’s t-test was adopted for group measurement data. The comparisons among groups were performed using the Chi squared test. For data that did not meet the requirements for the Chi squared test, Fisher’s exact probability test was used. Data that were not normally distributed were compared by the Mann-Whitney U test. All tests were two-tailed, and p-values < 0.05, p-values < 0.01 were considered statistically significant.

## Additional Information

**How to cite this article**: Wang, Y. *et al.* Clinical characteristics of protracted bacterial bronchitis in Chinese infants. *Sci. Rep.* **5**, 13731; doi: 10.1038/srep13731 (2015).

## Figures and Tables

**Table 1:** Characteristics and clinical features of patients with PBB and no PBB controls.

Characteristics	PBB group (n = 50), (%)	No PBB group (n = 16), (%)	p
mean age [months]	10.00 (5.75–14)	12 (7–18.5)	>0.05
gender			
male	43 (86)	12 (75)	>0.05
female	7 (14)	4 (25)	>0.05
symptoms			
wet cough	50 (100)	16 (100)	>0.05
wheezing	45 (90)	10 (62.5)	<0.01***
stridor	2 (4)	1 (6.25)	>0.05
phase of cough			
morning	8 (16)	4 (25)	>0.05
night	8 (16)	4 (25)	>0.05
both day and night	32 (64)	7 (43.8)	<0.05*
post-activity	2 (4)	1 (6.25)	>0.05
median duration of cough [weeks]	10.0 ± 16.4	15.1 ± 1 6.3	>0.05
physical examination			
wheezing rales	41 (82)	11 (68.7)	>0.05
moist rales	23 (46)	9 (56.2)	>0.05

P-values < 0.05* and <0.01*** were considered statistically significant.

**Table 2:** Demographic and clinical features in PBB with or without airway deformity.

Characteristics	no airway deformity (n = 28), n (%)	airway deformity (n = 22) n (%)	p
mean age [months]	12 (7.25–16.75)	8 (5–10)	<0.05*
gender			
male	23 (82.1)	20 (90.9)	>0.05
female	5 (17.9)	2 (9.1)	>0.05
symptom			
cough	28 (100)	22 (100)	>0.05
wheezing	23 (82.1)	20 (90.9)	>0.05
stridor	0 (0)	2 (28.6)	<0.05*
phase of cough			
in the morning	6 (21.4)	2 (9.1)	>0.05
at night	5 (17.9)	3 (13.6)	>0.05
both day and night	17 (60.7)	15 (68.2)	>0.05
post-activity	0 (0)	2 (9.1)	>0.05
median duration of cough [days]	9.1 ± 13.7	11.3 ± 19.6	>0.05
physical examination			
lung wheezing rales	23 (82.1)	18 (79.6 )	>0.05
lung moist rales	11 (39.2)	12 (9.3)	>0.05
cellularity of BALF			
neutrophil (%)	26.0 ± 32.15	33.3 ± 27.6	>0.05
lymphocyte (%)	7.2 ± 6.4	9.1 ± 6.6	>0.05
macrophage (%)	64.2 ± 34.2	56.9 ± 31.0	>0.05
eosinophil (%)	2.6 ± 13.2	0.60 ± 2.0	>0.05
positive bacterial cultures of BALF	11 (39.3)	8 (36.4)	>0.05
Streptococcus pneumoniae	5 (17.9)	4 (18.2)	>0.05
Haemophilus influenzae	5 (17.9)	1 (4.5)	>0.05
E. coli	1 (3.6)	1 (4.5)	>0.05
Andenteroaerogen	0 (0)	1 (4.5)	>0.05
Filamentous fungi	0 (0)	1 (4.5)	>0.05
immunoglobulins (g/l)			
IgA	0.3 ± 0.3	0.3 ± 0.3	>0.05
IgG	5.4 ± 2.0	6.0 ± 2.6	>0.05
IgM	1.2 ± 0.6	0.9 ± 0.4	>0.05
lymphocyte subsets (%)			
CD3^+^	60.0 ± 10.7	58.3 ± 13.5	>0.05
CD3^+^CD4^+^	35.1 ± 9.1	34.2 ± 12.5	>0.05
CD4^+^CD8^+^	22.0 ± 7.6	21.9 ± 7.6	>0.05
CD3^−^CD19^+^	29.1 ± 10.9	26.8 ± 11.4	>0.05
CD3^−^CD16^+^CD56^+^	9.0 ± 6.3	12.9 ± 9.1	>0.05
CD19^+^CD23^+^	14.0 ± 6.3	13.6 ± 6.1	>0.05

P-values < 0.05* were considered statistically significant.

**Table 3:** BALF cytology and immune function of patients with PBB and controls.

Characteristics	PBB group (n = 50), n (%)	No PBB group (n = 16), n (%)	p
cellularity of BALF			
neutrophil (%)	29.2 ± 30.2	2.9 ± 3.5	<0.01**
lymphocyte (%)	8.1 ± 6.5	8.4 ± 4.3	>0.05
macrophage (%)	61.0 ± 32.7	88.7 ± 3.6	<0.01**
eosinophil (%)	1.7 ± 9.9	0.00 ± 0.00	>0.05
positive bacterial cultures of BALF	19 (38)	0 (0)	<0.05*
airway deformity	22 (44)	10 (62.5)	>0.05
immunoglobulins (g/L)			
IgA	0.3 ± 0.3	0.5 ± 0.6	>0.05
IgG	5.65 ± 2.3	6.0 ± 2.2	>0.05
IgM	1.1 ± 0.6	1.2 ± 0.7	>0.05
lymphocyte subsets (%)			
CD3^+^	59.3 ± 11.9	68.6 ± 4.25	<0.01**
CD3^+^CD4^+^	34.7 ± 10.6	42.5 ± 6.8	<0.01**
CD4^+^CD8^+^	1.9 ± 1.3	2.1 ± 1.0	>0.05
CD3^−^CD19^+^	28.1 ± 11.1	23.2 ± 5.05	<0.05*
CD3^−^CD16^+^CD56^+^	10.75 ± 7.8	7.45 ± 3.7	<0.05*
CD19^+^CD23^+^	13.9 ± 6.2	5.4 ± 2.65	<0.01**

P-values < 0.05*, <0.01** were considered statistically significant.

**Table 4:** Associations between lymphocyte subsets and cough severity in PBB patients.

Characteristic	VCD scores ≥3 (n = 25)	VCD scores 1~2 (n = 21)	VCD scores 0 ~1 (n = 4)	p
lymphocyte subsets (%)				
CD3^+^	58.55 ± 12.95	62.37 ± 12.69	65.10 ± 12.30	>0.05
CD3^+^CD4^+^	35.28 ± 11.18	38.98 ± 12.87	31.80 ± 2.54	>0.05
CD4^+^CD8^+^	20.63 ± 5.44	20.70 ± 9.57	31.60 ± 13.15	>0.05
CD3^−^CD19^+^	28.71 ± 12.22	26.89 ± 13.63	19.40 ± 20.51	>0.05
CD3^−^CD16^+^CD56^+^	11.01 ± 6.24	8.74 ± 8.47	14.10 ± 8.49	>0.05
CD19^+^CD23^+^	13.41 ± 5.57	12.51 ± 7.76	14.00 ± 16.97	>0.05

VCD, verbal category descriptive.

**Table 5:** Associations between lymphocyte subsets and cough duration in PBB patients.

Characteristic	4-8 weeks (n = 33)	8-12 weeks (n = 8)	≥12 weeks (n = 9)	p
lymphocyte subsets (%)				
CD3^+^	60.15 ± 11.82	57.20 ± 3.25	60.83 ± 6.99	>0.05
CD3^+^CD4^+^	35.26 ± 10.14	33.30 ± 4.24	35.03 ± 5.65	>0.05
CD4^+^CD8^+^	21.73 ± 8.38	21.80 ± 0.28	23.45 ± 4.99	>0.05
CD3^−^CD19^+^	29.44 ± 11.43	21.60 ± 10.47	31.35 ± 9.34	>0.05
CD3^−^CD16^+^CD56^+^	8.69 ± 5.73	19.50 ± 7.07	5.65 ± 3.82	>0.05
CD19^+^CD23^+^	13.71 ± 6.56	11.45 ± 0.50	17.10 ± 6.30	>0.05
